# A meta-analysis on the effects of probiotics on the performance of pre-weaning dairy calves

**DOI:** 10.1186/s40104-022-00806-z

**Published:** 2023-01-04

**Authors:** Liyun Wang, Honghong Sun, Haixu Gao, Yaohui Xia, Linsen Zan, Chunping Zhao

**Affiliations:** College of Animal Science and Technology, Northwest A&F University, No.22 Xinong Road, Yangling, Shaanxi 712100 P. R. China

**Keywords:** Calves, Growth, Health, Meta-analysis, Probiotics

## Abstract

**Background:**

Probiotics have been used in livestock production for many years, but information on their benefits during the early life of calves is inconsistent. This study aimed to assess the effects of probiotics on the performance of pre-weaning dairy calves and identify the factors influencing their effect sizes.

**Results:**

Forty-nine studies were selected for meta-analysis based on the inclusion and exclusion criteria. The study qualities were evaluated using a predefined risk assessment tool following GRADE guidelines. Meta-analysis results showed that probiotics increased the growth performance (body weight by 1.988 kg and average daily gain by 40.689 g/d), decreased digestibility and feed efficiency (feed conversion rate by 0.073), altered rumen parameter (decreased acetate by 2.815 mmol/L and increased butyrate by 0.788 mmol/L), altered blood parameter (decreased AST by 4.188 U/L, increased BHBA by 0.029 mmol/L and IgG by 0.698 g/L), increased faecal parameter (faecal bacteria counts by 0.680 log_10_ CFU/g), based on the strict criteria (*P*_SMD_ < 0.05, *I*^*2*^ < 50%). Additionally, probiotics increased digestibility and feed efficiency (starter dry matter intake by 0.034 kg/d and total dry matter intake by 0.020 kg/d), altered blood parameter (increased IgA by 0.313 g/L, IgM by 0.262 g/L, and total antioxidant capacity by 0.441 U/mL, decreased MDA by 0.404 nmol/mL), decreased faecal parameter (faecal score by 0.052), based on the loose criteria (*P*_SMD_ < 0.05, *I*^*2*^ > 50%).

Regression and sub-group analyses showed that probiotic strains, supplementation dosage, and methods significantly affected the performance of calves. The probiotics supplied with more than 9.5 log_10_ CFU/d significantly increased IgA and IgM contents (*P*_SMD_ < 0.05). Additionally, the compound probiotics significantly increased TDMI, IgA, and IgM (*P*_SMD_ ≤ 0.001). Furthermore, probiotics supplemented in liquid (whole milk or milk replacer) significantly increased TDMI and decreased faecal score (*P*_SMD_ < 0.05), while in whole milk, they significantly increased body weight, IgA, and IgM (*P*_SMD_ < 0.001).

**Conclusions:**

Probiotics could improve the growth performance, feed intake and efficiency, rumen fermentation, immune and antioxidant capacity, and health of pre-weaning calves. However, the effect sizes were related to the dosage, composition, and supplementation methods of probiotics.

**Supplementary Information:**

The online version contains supplementary material available at 10.1186/s40104-022-00806-z.

## Introduction

Most modern intensive rearing systems for dairy cows require that the calves are immediately separated from dams after birth and then artificially fed on whole milk or milk replacer. As a result, the newborn calves cannot rapidly acquire microflora from the saliva and feces of their mothers and other cows. This slows the formation of microbial communities and can even cause an imbalanced microbial flora in the digestive tracts of the calves [1]. Further, it may negatively affect the growth rate and health status of calves and even production performance if proper feeding and management strategies are not adopted during this critical life stage [2]. Feed additives have been extensively studied and commercially explored in livestock production. Probiotics are one of the most popular feed additives due to their beneficial effect on domestic animals [3]. Probiotics are live microorganisms that modulate the balance and activities of the gastrointestinal microbiota and thus can enhance the host’s health and growth if administered in adequate dosages [4].

Numerous studies have examined the effect of probiotics on the production performance and health of dairy calves [5]. However, the results are inconsistent and even contradictory. Some studies have shown that probiotics can promote total dry matter intake (TDMI), body weight (BW), and growth rate of calves [5, 6]; others have concluded that probiotics do not affect the growth performance or feed efficiency [7, 8]. Additionally, some studies have found that probiotics can increase blood IgA, IgG, and IgM concentrations and correspondingly improve the immunity of calves [9]; others observed no effect of probiotics on the concentration of these three immune globulins [10]. Furthermore, some studies have identified that probiotics can reduce faecal scores of calves [11, 12]; other reported no differences on faecal scores by adding probiotics [13]. Meta-analysis has been applied in animal science for systemic evaluation of the effect of probiotics on calves. A meta-analysis reported that probiotics could increase the average daily gains (ADG) by 83.14 g/d and decrease the feed conversion ratio (FCR) by 0.13 compared with calves fed on a control diet, but it did not sufficiently show the effect of probiotics on calf health [7]. Additionally, a meta-analysis included dairy calves (Holstein and Jersey), beef calves (Charolais and Red Angus), other cross-bred cattle, local cattle breeds (Qinchuan cattle in China and Hanwoo in Korea), Bubalus bubalis, and Murrah buffalo, but dairy calves had a very different rearing and management system from others, especially in the pre-weaning stage. Therefore, it could not accurately reflect the probiotics function on the pre-weaning dairy calves [7]. In another meta-analysis including 15 trials, supplementation of probiotics reduced the relative risk of diarrhea and feeding in the whole milk improved the protective effect [14]. However, it did not include other production performance and needs to update due that it has been done 10 years ago.

In this study, we hypothesized that probiotics could improve the growth rate, digestibility, immune, and health of dairy calves across the published studies. This study aimed to critically review the studies and enhance understanding of the effects of probiotics on pre-weaning dairy calves. The existence of heterogeneity and its sources were also assessed. Therefore, this study will provide insights into establishing proper feeding and management strategies for efficient application of probiotics in calf rearing.

## Methods

### Literature search strategy and selection criteria

This study was based on the Preferred Reporting Items for Systematic Reviews and Meta-analysis Statement [15]. Three researchers independently searched PubMed, ScienceDirect, Web of Science, and Google Scholar (before Jan 10, 2022) using the MeSH terms “probiotics, or any name of the species or strain of probiotics, in combination with “calf” or “calves” to identify eligible studies.

### Inclusion and exclusion criteria

Search results from the four databases were pooled in EndNote (Version X9) and then duplicate publications were removed. Literature was rigorously screened based on the inclusion and exclusion criteria. Differences were resolved through discussions. The references from the search were included or excluded based on the following criteria: Inclusion criteria: (1) manuscripts published in English in peer-reviewed journals, (2) studies involving the use of probiotics in the diet of dairy calves; (3) studies including probiotics treatment and negative control groups; (4) studies with continuous experiment rather than Latin square or change-over designs; (5) studies providing adequate probiotics data, the number of cattle, mean, standard deviation or standard error of at least one of the traits corresponding to the probiotics group and control group. The exclusion criteria included: (1) studies without probiotics data or correlated traits data; (2) studies with the data from non-dairy cattle; (3) studies with post-weaning calves; (4) studies with probiotics combined with prebiotics or antibiotics.

### Data extraction

The following variables: first author, year, country, calf breed, age, sample size, probiotic composition, experiment duration, diet composition, supplementation methods, mean, standard deviation or standard error of all traits corresponding to probiotics and control groups, were extracted from each study. The main traits were in five categories: growth performance (BW, ADG, withers height, heart girth, hip width, hip height, and body length); feed digestibility and efficiency (organic matter digestibility, dry matter digestibility, ether extract digestibility, crude protein digestibility, NDF digestibility, ADF digestibility, TDMI, SDMI, and FCR (TDMI/ADG)); rumen parameter (rumen pH, microbial protein, NH_3_, total volatile fatty acids (VFA), acetate, propionate, butyrate, and valerate); haematology parameter (biochemical indexes, such as alkaline phosphatase (ALP), albumin (ALB), alanine aminotransferase (ALT), aspartate aminotransferase (AST), beta-hydroxybutyric acid (BHBA), blood urea nitrogen (BUN), glucose, total protein, total cholesterol, triglyceride, and lactate dehydrogenase (LDH)); immune indices (immunoglobulin A (IgA), immunoglobulin G (IgG), immunoglobulin M (IgM), insulin-like growth factor 1 (IGF1), and interferon-γ (IFNγ)); antioxidant indices (malondialdehyde (MDA), glutathione peroxidase (GSH-Px), superoxide dismutase (SOD), and total antioxidant capacity (T-AOC)); faecal parameter (faecal score, count of faecal bacteria, coliform, *Lactobacilli*, and *Streptococcus*).

The possible sources of variability, calf ages, BW at the beginning and end of the experiment, additive dosage, supplementation methods, and experiment duration were also extracted from each study.

### Study quality assessment

Two researchers independently assessed the study quality following the criteria in the Cochrane Collaboration’s tool and the statement of Consolidated Standards of Reporting Trials [16, 17]. The assessment items included random sequence generation (selection bias), allocation concealment (selection bias), blinding of participants and personnel (performance bias), blinding of outcome assessment (detection bias), incomplete outcome data (attrition bias), selective reporting (reporting bias), and other bias. The disagreements on assessment were settled by discussions with a third researcher.

### Statistical analysis

#### Meta-analysis

Meta-analysis was performed using the STATA/MP 14.0 software (version 11.0, College Station, TX). The random-effects model was used to estimate the effect size, 95% confidence interval (CI), and statistical significance for each trait since it is more conservative than the fixed-effects model [18, 19]. The effect size of probiotics was expressed as standard mean difference (SMD) and raw mean difference (RMD). The SMD showed the effect size in standard deviation unit and was more generalizable, while RMD expressed the effect size in the same unit as the original measurement and was more interpretable [20]. SMD values of < 0.2, 0.2 < SMD < 0.7, and > 0.7 indicated small, moderate, and high effects, respectively [20, 21]. A *P*-value of SMD less than 0.05 was considered statistically significant.

#### Heterogeneity assessment

Heterogeneity between-study variability was assessed using the *I*^*2*^ (*I*^*2*^ < 25% = no heterogeneity, 25% ≤ *I*^*2*^ < 50% = moderate heterogeneity, 50% ≤ *I*^*2*^ < 75% = high heterogeneity, 75% ≤ *I*^*2*^ < 100% = extreme heterogeneity) [22]. Meta-regression or sub-group analysis was necessary to further determine the sources of heterogeneity when the studies had a substantial heterogeneity (*I*^*2*^ > 50%) [22].

#### Meta-regression analysis

Meta-regression analyses were conducted using effect sizes (RMD) for each outcome (*P*_SMD_ < 0.05, *I*^*2*^ > 25%, *n* ≥ 10) as the dependent variable to examine heterogeneity sources of meta-analysis. The covariates included the calf age (d), beginning BW (kg), probiotic composition (single strain/multiple strains), dosage (log_10_ CFU/d), supplementation methods (milk, replacer, starter), and experiment duration (d). Tests of the null hypothesis for the covariate coefficients were obtained from the modified Knapp-Hartung method. The adjusted R^2^ represented the proportion of between-study variation explained by the covariates [23]. The RMDs were measured via sub-group analysis if the *P*-value of covariates in meta-regression was less than 0.05. The sub-groups were divided based on the original categories and practical implications where necessary.

#### Publication bias

Begg’s and Egger’s tests were used to assess publication bias. A *P*-value less than 0.05 was defined as significant [24, 25]. Egger’s test was first adopted if the significant tests disagreed using both methods [26].

## Results

The process and results of the literature search and selection are shown in Fig. S1. Initially, 7033 articles were identified for screening from PubMed, ScienceDirect, Web of Science, Google Scholar, and other sources. A total of 657 articles remained after excluding duplication, articles with non-dairy cattle, post-weaning calves, feeding probiotics mixed with other additives, published in non-English, or with no corresponding production traits. An additional 506 articles were excluded after full-text review based on previous protocols. Finally, 49 articles were included in this meta-analysis.

The main characteristics of the 49 studies are shown in Table S1. The bias risks for each study and overall are shown in Fig. S2 and S3. Selection bias (random sequence generation and allocation concealment), attrition bias, and reporting bias were at low risk in over 75% of the studies. Performance and detection biases were largely unclear in over 90% of the studies due to insufficient information on the blinding of participants and reporting. Descriptive statistics for the five categories of variables are shown in Table S2.

### Growth performance traits

The summary of the meta-analysis on the effects of probiotics on the growth performance of pre-weaning calves is shown in Table 1. Probiotics did not significantly affect withers height, heart girth, hip width, hip height, and body length (*P*_SMD_ > 0.05). However, probiotics significantly increased BW (SMD = 0.315, *P* < 0.001, *I*^*2*^ = 41.2%) and ADG (SMD = 0.374, *P* < 0.001, *I*^*2*^ = 39.0%), indicating moderate effect (0.2 < SMD < 0.7). Correspondingly, the RMD analysis showed that probiotics increased BW and ADG by 1.988 kg and 40.689 g/d, respectively.

**Table 1:** The summary of the meta-analysis on the effects of probiotics on the growth performance of pre-weaning calves

		SMD			Heterogeneity			RMD				Egger’s test	Begg’s test
	*n*	Random effect	95% CI	*P*-value	Chi-squared (Q)	*P*-value	*I*^*2*^, %	Control mean	Random effect	95% CI	*P*-value	*P*-value	*P*-value
Withers height, cm	26	0.128	−0.169, 0.425	0.397	106.650	< 0.001	76.6	82.433	0.507	−0.538, 1.553	0.342	0.005	0.038
Heart girth, cm	25	0.095	−0.149, 0.339	0.447	63.110	< 0.001	62.0	92.334	0.270	−0.454, 0.994	0.464	0.005	0.072
Hip width, cm	7	0.244	−0.093, 0.581	0.156	9.860	0.131	39.2	20.950	0.377	−0.132, 0.885	0.146	0.049	0.133
Hip height, cm	14	−0.067	−0.486, 0.353	0.756	52.150	< 0.001	75.1	87.649	0.148	−0.956, 1.251	0.793	0.274	0.584
Body length, cm	11	−0.401	−0.821, 0.020	0.062	31.200	0.001	67.9	86.987	−1.149	−2.274, −0.024	0.045	0.078	0.350
BW, kg	62	0.315	0.188, 0.441	< 0.001	103.770	0.001	41.2	73.498	1.988	1.173, 2.803	< 0.001	0.090	< 0.001
ADG, g/d	79	0.374	0.275, 0.473	< 0.001	127.960	< 0.001	39.0	505.731	40.689	30.881, 50.496	< 0.001	0.052	0.002

Meta-regression analysis showed that per kilogram of beginning BW of calves increased the final BW by 0.312 kg (*P* < 0.001). The supplementation methods increased the final BW by 1.576 kg (*P* = 0.002) (Table 6). Sub-group analysis indicated that probiotics supplied in whole milk significantly increased the final BW by 4.439 kg (*P* < 0.001, *I*^*2*^ = 31.0%) (Table 7). The Egger’s and Begg’s tests results indicated no evidence of publication bias in the two growth traits (*P* > 0.05) (Table 1).

### Feed digestibility and efficiency

The summary of the meta-analysis on the effects of probiotics on the feed digestibility and efficiency of pre-weaning calves is shown in Table 2. Probiotics did not significantly affect the digestibility of organic matter, dry matter, ether extract, crude protein, neutral detergent fiber, and acid detergent fiber (*P*_SMD_ > 0.05). However, probiotics significantly increased SDMI (SMD = 0.439, *P* < 0.001, *I*^*2*^ = 64.2%) and TDMI (SMD = 0.329, *P* = 0.004, *I*^*2*^ = 69.5%), while they decreased FCR (SMD = −0.305, *P* < 0.001, *I*^*2*^ = 32.1%), indicating moderate effect. Correspondingly, the RMD analysis showed that probiotics increased SDMI and TDMI by 0.034 kg/d and 0.020 kg/d, respectively, while decreased FCR by 0.073.

**Table 2:** The summary of the meta-analysis on the effects of probiotics on the feed digestibility and efficiency traits of pre-weaning calves

		SMD			Heterogeneity			RMD				Egger’s test	Begg’s test
	*n*	Random effect	95% CI	*P*-value	Chi-squared (Q)	*P*-value	*I*^*2*^, %	Control mean	Random effect	95% CI	*P*-value	*P*-value	*P*-value
Organic matter digestibility, %	8	0.242	−0.138, 0.623	0.211	1.480	0.983	0	86.400	0.724	−0.924, 2.372	0.389	0.282	0.386
Dry matter digestibility, %	8	0.332	−0.049, 0.713	0.087	0.630	0.999	0	84.240	1.195	−0.368, 2.757	0.134	0.955	0.386
Ether extract digestibility, %	5	0.324	−0.149, 0.796	0.179	0.230	0.994	0	83.800	1.587	−1.509, 4.684	0.315	0.059	1.000
Crude protein digestibility, %	7	0.290	−0.113, 0.692	0.158	3.240	0.779	0	82.986	1.339	−0.613, 3.291	0.179	0.893	1.000
NDF digestibility, %	2	3.828	−6.266, 13.921	0.457	8.550	0.003	88.3	54.900	8.808	−10.889, 28.506	0.381		1.000
ADF digestibility, %	3	0.134	−0.796, 1.065	0.777	3.940	0.140	49.2	57.200	0.711	−2.717, 4.138	0.684	0.518	1.000
SDMI, kg/d	42	0.439	0.234, 0.644	< 0.001	114.640	< 0.001	64.2	2.655	0.034	0.014, 0.053	0.001	0.030	0.050
TDMI, kg/d	47	0.329	0.105, 0.552	0.004	150.820	< 0.001	69.5	1.003	0.020	0.006, 0.033	0.005	0.025	0.137
FCR, kg/kg	35	−0.305	−0.466, −0.144	< 0.001	50.090	0.037	32.1	2.467	−0.073	−0.115, −0.031	0.001	0.012	0.005

Meta-regression analysis showed that per kilogram of beginning BW of calves tended to increase TDMI by 0.052 kg/d (*P* = 0.096). In contrast, probiotic strains tended to decrease TDMI by 0.032 kg/d (*P* = 0.074). Additionally, the supplementation methods significantly increased the TDMI by 0.019 kg/d (*P* = 0.045) (Table 6). Sub-group analysis showed that although compound probiotics significantly increased the TDMI by 0.028 kg/d (*P* = 0.001), heterogeneity was still high (*I*^*2*^ = 76.9%) (Table 7). Furthermore, probiotics supplied in the starter did not affect TDMI (*P* > 0.05). However, probiotics supplied in whole milk and milk replacer significantly increased TDMI by 0.033 kg/d (*P* = 0.018, *I*^*2*^ = 87.0%) and 0.019 kg/d (*P* = 0.030, *I*^*2*^ = 64.3%), respectively. The Egger’s and Begg’s tests indicated some evidence of publication bias in SDMI, TDMI, and FCR (*P* < 0.05) (Table 2).

### Rumen parameter

The summary of the meta-analysis on the effects of probiotics on the rumen fermentation parameters of pre-weaning calves is shown in Table 3. Probiotics did not significantly affect rumen pH, microbial protein, NH_3_, total VFA, propionate, and valerate (*P*_SMD_ > 0.05). However, probiotics significantly decreased acetate (SMD = −0.453, *P* = 0.016, *I*^*2*^ = 36.2%) and increased butyrate (SMD = 0.722, *P* < 0.001, *I*^*2*^ = 49.0%), indicating moderate effect on acetate and high effect on butyrate. Correspondingly, the RMD analysis showed that probiotics decreased acetate by 2.815 mmol/L and increased butyrate by 0.788 mmol/L.

**Table 3:** The summary of the meta-analysis on the effects of probiotics on the rumen fermentation parameters of pre-weaning calves

		SMD			Heterogeneity			RMD				Egger’s test	Begg’s test
	*n*	Random effect	95% CI	*P*-value	Chi-squared (Q)	*P*-value	*I*^*2*^, %	Control mean	Random effect	95% CI	*P*-value	*P*-value	*P*-value
Rumen pH	20	0.412	−0.171, 0.995	0.166	137.78	< 0.001	86.9	5.958	0.154	0.128, 0.180	< 0.001	0.009	0.014
Microbial protein, mg/dL	2	2.173	−0.975, 5.321	0.176	15.29	< 0.001	93.5	163.620	13.184	10.457, 15.912	< 0.001		1.000
NH_3_, mmol/L	17	−0.103	−0.482, 0.276	0.595	47.55	< 0.001	66.4	7.924	−0.113	−0.830, 0.604	0.757	0.228	0.138
Total VFA, mmol/L	13	0.053	−0.538, 0.643	0.861	63.37	< 0.001	81.1	71.916	−0.271	−3.238, 2.696	0.858	0.006	0.017
Acetate, mmol/L	10	−0.453	−0.822, −0.084	0.016	14.10	0.119	36.2	40.463	−2.815	−4.946, −0.684	0.010	0.477	0.788
Propionate, mmol/L	12	0.168	−0.740, 1.075	0.718	102.28	< 0.001	89.2	26.808	−0.44	−2.861, 1.981	0.722	0.094	0.216
Butyrate, mmol/L	15	0.722	0.356, 1.088	< 0.001	27.43	0.017	49.0	4.823	0.788	0.307, 1.270	0.001	0.570	0.234
Valerate, mmol/L	15	−0.174	−0.772, 0.423	0.568	97.21	< 0.001	85.6	1.739	−0.055	−0.127, 0.016	0.130	0.288	0.692

Meta-regression analysis showed that none of the six covariates was a significant source of heterogeneity for acetate. However, the supplementation dosage was the source of heterogeneity for butyrate (*P* = 0.039) (Table 6). Sub-group analysis indicated that probiotics supplied at the high dosage (> 10 log_10_ CFU/d) significantly increased butyrate by 0.463 mmol/L (*P* = 0.013, *I*^*2*^ = 47.8%), but those at the low dosage (< 9 log_10_ CFU/d) had high heterogeneity (*I*^*2*^ = 69.9%) (Table 7). The Egger’s and Begg’s tests indicated no evidence of publication bias in the two traits (*P* > 0.05) (Table 3).

### Haematology parameter

The summary of the meta-analysis on the effects of probiotics on the blood biochemistry, immunity, and antioxidant indices of pre-weaning calves is shown in Table 4. Probiotics did not significantly affect some biochemical indexes (ALP, ALB, ALT, BUN, Glucose, total protein, total cholesterol, triglyceride), immune indices (IGF1 and IFNγ), and antioxidant indices (GSH-Px and SOD) (*P*_SMD_ > 0.05). However, probiotics significantly influenced some biochemical indexes, including AST (*P*_SMD_ = 0.001, RMD = −4.188 U/L, *I*^*2*^ = 44.6%), BHBA (*P*_SMD_ = 0.044, RMD = 0.029 mmol/L, *I*^*2*^ = 27.6%), and LDH (*P*_SMD_ < 0.001, RMD = −78.796 U/L, *I*^*2*^ = 52.7%), immune indices, including IgA (*P*_SMD_ < 0.001, RMD = 0.313 g/L, *I*^*2*^ = 68.4%), IgG (*P*_SMD_ < 0.001, RMD = 0.698 g/L, *I*^*2*^ = 28.5%), IgM (*P*_SMD_ < 0.001, RMD = 0.262 g/L, *I*^*2*^ = 68.7%), and antioxidant indices, including MDA (*P*_SMD_ = 0.027, RMD = −0.404 nmol/ml, *I*^*2*^ = 80.5%) and T-AOC (*P*_SMD_ = 0.016, RMD = 0.441 U/mL, *I*^*2*^ = 65.1%).

**Table 4:** The summary of the meta-analysis on the effects of probiotics on the blood biochemistry, immunity, and antioxidant indices of pre-weaning calves

		SMD			Heterogeneity			RMD				Egger’s test	Begg’s test
	*n*	Random effect	95% CI	*P*-value	Chi-squared (Q)	*P*-value	*I*^*2*^, %	Control mean	Random effect	95% CI	*P*-value	*P*-value	*P*-value
Biochemical indexes													
ALP, U/L	8	0.554	−0.231, 1.339	0.166	36.490	< 0.001	80.8	134.004	4.368	−3.901, 12.637	0.301	0.037	0.266
ALB, g/L	9	0.751	−0.124, 1.627	0.093	50.100	< 0.001	84.0	29.402	0.473	−0.205, 1.151	0.172	0.001	0.251
ALT, U/L	7	0.476	−0.597, 1.549	0.385	46.600	< 0.001	87.1	13.556	0.237	−0.619, 1.093	0.587	0.235	0.548
AST, U/L	8	−0.792	−1.243, −0.342	0.001	12.650	0.081	44.6	51.416	−4.188	−6.741, −1.635	0.001	0.069	0.035
BHBA, mmol/L	7	0.419	0.011, 0.826	0.044	8.290	0.217	27.6	0.257	0.029	−0.003, 0.062	0.077	0.533	0.548
BUN, mmol/L	14	0.162	−0.998, 1.321	0.785	172.060	< 0.001	92.4	3.484	0.056	−0.245, 0.358	0.714	0.748	0.827
Glucose, mmol/L	20	−0.274	−0.828, 0.280	0.333	123.790	< 0.001	84.7	4.219	−0.055	−0.236, 0.125	0.548	0.081	0.074
Total protein, g/L	20	0.260	−0.110, 0.630	0.169	63.780	< 0.001	70.2	56.384	0.750	−0.506, 2.007	0.242	0.492	0.347
Total cholesterol, mmol/L	12	−0.731	−1.834, 0.373	0.194	121.270	< 0.001	90.9	2.224	−0.060	−0.387, 0.268	0.720	0.803	0.304
Triglyceride, mmol/L	5	−0.760	−2.270, 0.749	0.323	40.950	< 0.001	90.2	0.309	−0.018	−0.051, 0.016	0.298	0.534	1.000
LDH, U/L	6	−1.359	−1.990, −0.728	< 0.001	10.570	0.061	52.7	708.462	−78.796	−123.059, −34.533	< 0.001	0.159	0.060
Immune indices													
IgA, g/L	24	0.806	0.468, 1.144	< 0.001	72.700	< 0.001	68.4	2.847	0.313	0.209, 0.418	< 0.001	< 0.001	< 0.001
IgG, g/L	26	0.371	0.165, 0.578	< 0.001	34.970	0.089	28.5	17.123	0.698	0.300, 1.095	0.001	0.073	0.011
IgM, g/L	25	0.823	0.486, 1.160	< 0.001	76.600	< 0.001	68.7	2.010	0.262	0.162, 0.363	< 0.001	< 0.001	< 0.001
IGF1, ng/mL	7	0.872	−0.637, 2.381	0.258	89.830	< 0.001	93.3	59.386	4.338	−3.577, 12.253	0.283	0.001	0.035
IFN-γ, pg/mL	4	1.214	−0.262, 2.690	0.107	24.330	< 0.001	87.7	47.043	4.277	3.359, 5.195	< 0.001	0.379	0.089
Antioxidant indices													
MDA, nmol/mL	5	−1.238	−2.338, −0.138	0.027	20.550	< 0.001	80.5	4.796	−0.404	−0.786, −0.021	0.039	0.881	0.462
GSH-P*x*, U/mL	5	−0.076	−1.169, 1.016	0.891	23.690	< 0.001	83.1	145.008	−2.938	−15.692, 9.817	0.652	0.416	0.806
SOD, U/mL	9	0.199	−0.531, 0.929	0.592	50.610	< 0.001	84.2	124.680	1.825	−6.306, 9.956	0.660	0.574	0.466
T-AOC, U/mL	8	0.596	0.112, 1.079	0.016	20.030	0.005	65.1	8.009	0.441	0.208, 0.675	< 0.001	0.101	0.108

Meta-regression analysis for blood traits (*n* > 10) showed that increasing probiotics by per log_10_ dosage increased IgA by 0.545 g/L (*P* = 0.003) and IgM by 0.267 g/L (*P* = 0.018). Probiotic strains decreased IgA by 0.487 g/L (*P* = 0.004) and tended to decrease IgM by 0.395 g/L (*P* = 0.074) (Table 6). Sub-group analysis showed that probiotics supplied at the low dosage (< 9 log_10_ CFU/d) did not affect IgA and IgM (*P* > 0.05) (Table 7). However, the middle (9.5—9.9 log_10_ CFU/d) and high (> 10 log_10_ CFU/d) dosages significantly increased IgA and IgM (*P*_SMD_ < 0.05, respectively). Additionally, single probiotics did not affect IgA and IgM (*P* > 0.05) while compound strains increased IgA (*P*_SMD_ < 0.001, RMD = 0.471 g/L, *I*^*2*^ = 62.9%) and IgM (*P*_SMD_ < 0.001, RMD = 0.324 g/L, *I*^*2*^ = 73.2%). Furthermore, probiotics supplied in whole milk increased IgA (*P*_SMD_ < 0.001, RMD = 0.554 g/L, *I*^*2*^ = 42.2%) and IgM (*P*_SMD_ < 0.001, RMD = 0.408 g/L, *I*^*2*^ = 59.5%), while probiotics in milk replacer did not affect IgA and IgM (*P*_SMD_ > 0.05) (Table 7). The Egger’s and Begg’s tests indicated some evidence of publication bias in IgA and IgM (*P* < 0.05) (Table 4).

### Faecal quality and intestinal flora parameter

The summary of the meta-analysis on the effects of probiotics on the faecal quality and intestinal flora of pre-weaning calves is shown in Table 5. Probiotics did not significantly affect the counts (log_10_ CFU/g) of coliform, *Lactobacilli*, and *Streptococcus* (*P*_SMD_ > 0.05). However, probiotics significantly decreased faecal score (SMD = −0.383, *P* = 0.015, *I*^*2*^ = 78.4%) and increased faecal bacteria counts (SMD = 0.361, *P* = 0.002, *I*^*2*^ = 36.9%). Correspondingly, the RMD analysis showed that probiotics decreased the faecal score by 0.052 and increased faecal bacteria counts by 0.680 log_10_ CFU/g.

**Table 5:** The summary of the meta-analysis on the effects of probiotics on the faecal quality and intestinal flora of pre-weaning calves

		SMD			Heterogeneity			RMD				Egger’s test	Begg’s test
	*n*	Random effect	95% CI	*P*-value	Chi-squared (Q)	*P*-value	*I*^*2*^, %	Control mean	Random effect	95% CI	*P*-value	*P*-value	*P*-value
Faecal score	25	−0.383	−0.690, −0.076	0.015	111.050	< 0.001	78.4	1.455	−0.052	−0.124, 0.020	0.160	< 0.001	< 0.001
Faecal bacteria count, log_10_ CFU/g	12	0.361	0.130, 0.593	0.002	17.430	0.096	36.9	11.532	0.680	0.227, 1.133	0.003	0.051	0.304
coliform, log_10_ CFU/g	16	0.089	−0.117, 0.295	0.399	25.750	0.041	41.7	6.838	0.164	−0.125, 0.452	0.267	0.727	0.444
*Lactobacilli*, log_10_ CFU/g	23	0.180	−0.025, 0.384	0.085	45.300	0.002	51.4	6.212	0.161	−0.018, 0.340	0.077	< 0.001	0.006
*Streptococcus*, log_10_ CFU/g	6	−0.058	−0.264, 0.149	0.583	1.180	0.947	0	11.707	−0.130	−0.372, 0.112	0.292	0.256	0.024

Meta-regression analysis showed that per day of age decreased faecal score by 0.009 (*P* = 0.012). Moreover, each day of supplementation duration tended to decrease faecal score by 0.002 (*P* = 0.064) and supplementation methods significantly decreased faecal score by 0.09 (*P* = 0.036) (Table 6). Sub-group analysis showed that probiotics did not affect faecal score of young calves (age ≤ 5 d) (*P* > 0.05) but significantly decreased faecal score of older groups (age ≥ 10 d) (*P*_SMD_ = 0.010, RMD = −0.061) (Table 7). Additionally, supplementation in short term (≤ 28 d) did not affect faecal score (*P* > 0.05) while supplementation in long term (≥ 42 d) significantly decreased faecal score (*P*_SMD_ = 0.002, RMD = −0.070). Furthermore, supplementation in starter significantly increased faecal score (*P*_SMD_ = 0.017, RMD = 0.126, *I*^*2*^ = 0) while supplementation in liquid feed (whole milk or milk replacer) significantly decreased faecal score (*P* < 0.05) but heterogeneities were still high (*I*^*2*^ = 66.6% and 70.1%, respectively) (Table 7).

**Table 6:** The summary of the meta-regression analysis on the effects of probiotics on pre-weaning dairy calves

Dependent variable (Y, RMD)	Meta-regression parameter (*P*-value)								
	Intercept	Duration, d	Age, d	Beginning BW, kg	Dosage, log_10_ CFU/d	Probiotic strains	Methods	adj R^2^	No. of
BW, kg	−15.251	−0.003 (0.887)	−0.120 (0.244)	0.312 (< 0.001)	0.331 (0.387)	−0.581 (0.626)	1.576 (0.002)	5.38	55
TDMI, kg/d	−0.114	0.000 (0.776)	0.001 (0.213)	0.052 (0.096)	−0.009 (0.230)	−0.032 (0.074)	0.019 (0.045)	38.98	39
Butyrate, mmol/L	38.493	−0.005 (0.826)	0.052 (0.710)	−.084 (0.485)	−3.057 (0.039)	−0.651 (0.640)	−2.036 (0.238)	33.98	13
IgA, g/L	−1.450	−0.003 (0.371)	−0.008 (0.200)	−.049 (0.073)	0.545 (0.003)	−0.487 (0.004)	−0.317 (0.189)	91.99	20
IgM, g/L	−1.876	−0.000 (0.871)	−0.003 (0.603)	0.005 (0.800)	0.267 (0.018)	−0.395 (0.074)	−0.056 (0.755)	95.59	21
Faecal Score	0.523	−0.002 (0.064)	−0.009 (0.012)	−0.007 (0.464)	−0.019 (0.455)	0.129 (0.110)	−0.090 (0.036)	< 0	24

**Table 7:** The summary of the sub-group analysis on the effects of probiotics on pre-weaning dairy calves

			RMD			Heterogeneity	SMD
		*n*	Random effect	95% CI	*P*-value	*I*^*2*^, %	*P*-value
BW							
All trials		55	1.885	1.009, 2.761	< 0.001	45.5	< 0.001
Methods	Milk replacer	30	0.847	−0.127, 1.822	0.088	50.2	0.151
	starter	2	1.663	−0.378, 3.704	0.110	0.0	0.094
	Whole milk	23	4.439	2.581, 6.297	< 0.001	31.0	< 0.001
TDMI							
All trials		39	0.023	0.009, 0.037	0.001	73.5	0.001
Strains	Compound	24	0.028	0.011, 0.046	0.002	76.9	0.001
	Single	15	0.013	−0.013, 0.040	0.332	66.7	0.329
Methods	Milk replacer	20	0.019	0.001, 0.037	0.044	64.3	0.030
	starter	8	0.002	−0.021, 0.025	0.873	0.0	0.379
	Whole milk	11	0.033	0.007, 0.058	0.014	87.0	0.018
Butyrate							
All trials		13	0.828	0.312, 1.345	0.002	55.5	< 0.001
Dosage(log)	low (< 9)	5	2.762	0.161, 5.363	0.037	69.9	0.022
	high (> 10)	8	0.463	0.037, 0.889	0.033	47.8	0.013
IgA							
All trials		20	0.296	0.191, 0.400	< 0.001	73.8	< 0.001
Dosage(log)	low (< 9)	3	0.040	−0.083, 0.163	0.523	0.0	0.533
	middle (9.5–9.9)	11	0.219	0.093, 0.345	0.001	68.3	0.015
	high (> 10)	6	0.618	0.295, 0.942	< 0.001	74.3	< 0.001
Strains	Compound	15	0.471	0.283, 0.659	< 0.001	62.9	< 0.001
	Single	5	−0.000	−0.058, 0.057	0.987	64.0	0.925
Methods	Milk replacer	7	−0.017	−0.047, 0.013	0.268	0.0	0.441
	Whole milk	13	0.554	0.367, 0.741	< 0.001	42.2	< 0.001
IgM							
All trials		21	0.255	0.152, 0.358	< 0.001	72.9	< 0.001
Dosage(log)	low (< 9)	3	−0.037	−0.126, 0.052	0.420	0.0	0.424
	middle (9.5–9.9)	11	0.266	0.130, 0.402	< 0.001	69.1	< 0.001
	high (> 10)	7	0.400	0.218, 0.582	< 0.001	73.4	0.001
Strains	Compound	15	0.324	0.192, 0.457	< 0.001	73.2	< 0.001
	Single	6	0.255	0.152, 0.358	0.848	0.0	0.354
Methods	Milk replacer	7	−0.006	−0.031, 0.019	0.653	0.0	0.892
	Whole milk	14	0.408	0.317, 0.499	< 0.001	59.5	< 0.001
Faecal score							
All trials		24	−0.047	−0.083, −0.011	0.011	68.8	0.031
Age	young(≤ 5)	17	−0.030	−0.084, 0.024	0.275	70.3	0.266
	old(≥ 10)	7	−0.061	−0.105, −0.018	0.006	52.7	0.010
Duration	short (≤ 28)	12	0.012	−0.059, 0.082	0.747	36.6	0.626
	long (≥ 42)	12	−0.070	−0.113, −0.026	0.002	73.5	0.002
Methods	Milk replacer	14	−0.051	−0.089, 0.014	0.007	70.1	0.018
	starter	6	0.126	0.025, 0.227	0.014	0.0	0.017
	Whole milk	4	−0.158	−0.273, −0.043	0.007	66.6	0.029

## Discussion

### The implications of this meta-analysis

Ruminants, especially cattle, have rumen and ruminal microbes that can produce and supply energy, protein, VFA, and vitamins to their hosts [27]. About 90% of protein absorbed in the small intestine and 50% of the energy requirement of the host are provided by ruminal microbes [28]. Therefore, calves should be fed on a nutritional diet and reared in favorable conditions to promote rumen and ruminal microbe development and to maintain health and promote growth [3]. Many rearing and management strategies, such as supplementation with probiotics, have been introduced into dairy production. But the effectiveness of probiotics on pre-weaning dairy calves is inconsistent due to divergent experiment conditions. Therefore, this meta-analysis will systematically evaluate the effects of probiotics in improving the production and health of calves and identify the potential variables modulating the effect sizes. The results will allow cattle ranchers optimize the application of probiotics and maximize the benefit of calf production.

### The increase in production performance

In this meta-analysis, the effects of probiotics on pre-weaning calves (crucial stage for growth and production) were systematically evaluated. The results showed that additive probiotics increased the SDMI and TDMI. The diet components and fiber contents of starter significantly influence the development of rumen bacteria, specifically fibrolytic bacteria and the corresponding fermentation function of young calves [29]. Additionally, the diet provides the substrates available for rumen fermentation, and its fermenting products stimulate the development and function of rumen [30]. For instance, increasing SDMI can modify the rumen fermentation pattern, alter the proportion of VFA, and increase the concentration of butyrate [31]. Butyrate is more effectively produced from the fermentation of concentrate than from roughage, and it plays pivotal roles in stimulating the development of rumen mucosa [32]. Butyrate is absorbed and converted into BHBA after the starter is fed, fermented in rumen, and then released into the blood circulation [31]. Therefore, the BHBA concentration in serum may indicate the concentrate intake and rumen development [33]. So, this meta-analysis showed that probiotics increased butyrate in rumen fluid and BHBA in the serum of calves, probably due to the increased SDMI and TDMI.

Probiotics can promote the production and function of digestive enzymes, such as cellulase, amylase, protease, and others [5], balance and stabilize beneficial microbial ecosystem in the gastrointestinal tract [34, 35], and restore the gut microflora [35]. Supplementation of *Lactobacillus rhamnosus* in the pre-weaning period can increase the microbial diversity and alter the order of dominant bacteria and relative abundance of bacterial families in calf rumen, thus increasing VFA production [12]. Supplementary yeast culture decreases *Prevotella* and increases *Butyrivibrio* in rumen fluid, thus increasing butyrate production, length of papilla [36], and rumen weight [37]. Probiotics can also enhance mineral bio-availability, digestive capacity, and nutrient absorption [38, 39]. Therefore, the combination of above factors can increase ADG and final BW and decrease FCR in this meta-analysis.

### The improvement of calf health

Blood parameters were measured to evaluate the nutrition level, metabolic capacity, pathological change, immune status, antioxidant traits, health condition, etc*.* [40]. AST, as a key enzyme in amino acid metabolism, is a specific indicator of liver inflammation. Decreased AST levels indicate an improved liver function in calves [40]. LDH plays a crucial role in carbohydrate metabolism. Elevated serum LDH levels occur during tissue damage or liver disease. Therefore, elevated LDH can indicate common injuries and diseases [41]. MDA is produced through lipid peroxidation of polyunsaturated fatty acids. Therefore, MDA levels can be used as a marker of lipid peroxidation and free radicals to assess the damage to tissues and cells [42]. In this meta-analysis, probiotics significantly decreased the levels of AST, LDH, and MDA in serum, implying that probiotics relieve dysfunction of the liver in this crucial stage and improve calf health [40].

Additionally, probiotics stimulate immunity by increasing immunoglobulin and macrophagic activity [34, 39], competitively excluding enteric pathogens by colonizing several probiotics in the intestine [43], thus inducing an antioxidant effect and reducing inflammation [3]. Probiotics increase SOD concentration in calf blood and improve antioxidant capacity by terminating the chain reactions of free radicals or scavenging reactive oxygen species [8]. Additionally, faecal score can predict diarrhea in young animals [44]. The higher faecal score, the softer the feces, and the higher the rate of diarrhea. Supplementation of *Lactobacillus rhamnosus* reduces faecal scores in pre-weaning calves, indicating that probiotics have an antibiotic effect and can reduce or even exclude adherence of pathogens due to the adhesive properties [45, 46]. In this meta-analysis, probiotics increased the immunoglobulin (IgA, IgG, and IgM) and antioxidant capacity (T-AOC), decreased AST, LDH, MDA, and synergistically lowered the faecal score of calves, indicating decreased rate of diarrhea and improved health of calves.

### Regression analysis

Regression analysis showed that six of the outcomes were affected by at least one of the six covariates (*P* < 0.05) (Table 6). These covariates explained up to 38.98% and 33.98% of heterogeneity in TDMI and butyrate, respectively. Furthermore, they explained most of the heterogeneity in IgA (91.99%) and IgM (95.59%), but they did not explain the heterogeneity in BW (5.38%), faecal score (adj R^2^ < 0), and other outcomes (*P* > 0.05), indicating that other unknown dietary and management associated factors may influence the effect of probiotics on pre-weaning dairy calves.

### Supplementation dosage

Probiotics were supplied at the recommended dosages (7.570–10.397 log_10_ CFU/d) in the studies included in this meta-analysis, which are supposed to have a beneficial effect on calves. Although probiotics significantly affected 17 outcomes, heterogeneity of some outcomes was high. For instance, IgA, and IgG contents were positively associated with the dosage of probiotics in this regression analysis. Research found that high dosages of probiotics may improve the immune systems of calves [8]. Herein, our sub-group analysis further confirmed that a lower dosage of probiotics (< 9 log_10_ CFU/d) did not affect IgA and IgG contents (Table 7), while a higher dosage (> 9.5 log_10_ CFU/d) significantly increased IgA and IgG contents (*P* < 0.05). This may be due to the special gastrointestinal environment of newborn calves [47], and the actual contents, or the quality of probiotics was insufficient to show performance responses as the instruction described [46]. Therefore, sufficient dosages and superior quality of probiotics should be supplied for the desired effects in domestic production, especially in calf diets.

### Probiotics composition

Our regression analysis showed that probiotic strains significantly affected IgA, and tended to influence TDMI and IgM. Sub-group analysis showed that the compound of multiple strain probiotics significantly increased TDMI, IgA, and IgM (*P* < 0.001), while a single strain did not affect these three traits (Table 7). This is because probiotics might be species and strain-specific, and multiple strains of probiotics may enhance the advantages compared with a single strain due to their synergetic effects [48].

### Supplementation methods

Meta-analysis showed that probiotics significantly increased SDMI, TDMI, BW, IgA, and IgM, while decreasing faecal score. Sub-group analysis showed that probiotics supplementation in starters did not have a beneficial effect on TDMI and faecal score. In contrast, supplementation in liquid (whole milk or milk replacer) significantly and positively influenced TDMI and faecal score (*P* < 0.05) (Table 7). Additionally, probiotics supplied in whole milk, not in milk replacer, significantly affected BW, IgA, and IgM (*P* < 0.001) (Table 7). The supplementation methods significantly influenced the six outcomes probably due to difference in pathogen load between milk replacer and whole milk [5] or route of delivery (solid or liquid) [46]. Solids enter the rumen while liquid circumvents the rumen into the abomasum in the early life of calves [49]. The different effect could also be due to the divergent protein and fiber contents of the starter [29]. Furthermore, the diet of calves gradually transits from predominant liquids (milk or milk replacer) to a solid diet (starter) during weaning with increasing fermentable carbohydrates [50]. This transition can change the components of ruminal microbiomes, such as increasing Proteobacteria and Firmicutes*,* and decreasing Bacteroidetes phylum [51].

### The limitation of this meta-analysis

This meta-analysis has some limitations. Firstly, we only included peer reviewed publications in English and excluded unpublished data, conference proceedings, non-English studies. But a meta-analysis showed that the effect of yeast on milk yield of lactating dairy cows had less heterogeneity compared peer-reviewed studies to non-peer-reviewed reports and the effect sizes had no significant difference between these two groups [52]. Additionally, exclusion of non-English publications for meta-analysis did not change overall conclusions [53]. Secondly, the experiment details were not fully reported in many included studies, which resulted that the number of studies for some performances were small in regression and sup-group analysis. For experiment, 47 studies were included for TDMI in mate-analysis but only 39 had detail information on composition and supplementation methods for further sub-group analysis. Thirdly, we identified some sources of heterogeneity across studies but the heterogeneities were still large in some sub-group analysis. This may be due to the complication of feeding experiments and variations within and among studies were not totally eliminated. Our sub-group analysis showed that other unknown factors may influence the effect sizes of probiotics apart from these six ones [14]. Therefore, further experiment details are needed for less heterogeneity, consistent results, and more accurate and reliable conclusions.

## Conclusions

This meta-analysis demonstrated that probiotics supplementation could improve growth performance and feed efficiency, as indicated by the increased BW and ADG, and the decreased FCR due to increased TDMI and SDMI. Probiotics modified the ruminal fermentation, as indicated by the decreased acetate and increased faecal bacteria counts, butyrate, and corresponding BHBA. Probiotic supplementation improved the health of calves, as indicated by decreased AST, LDH, MDA, and faecal score and increased IgA, IgG, IgM, and T-AOC. The probiotics supplied with more than 9.5 log_10_ CFU/d distinctly improved IgA and IgM contents. Compound probiotics significantly affected TDMI, IgA, and IgM. Additionally, the supplementation methods significantly influenced SDMI, TDMI, BW, IgA, IgM, and faecal scores. These results further confirmed that probiotics supplementation could improve the growth, feed efficiency, and health of pre-weaning dairy calves. However, the effect sizes were related to the dosage, composition of strains, and supplementation methods. This meta-analysis addresses the controversy regarding the effect of probiotics on the pre-weaning of dairy calves and provides the fundamentals for the efficient use of probiotics in cattle production.

## Supplementary Information

**Additional file 1: Table S1.** Characteristics of the included studies in the meta-analysis. **Table S2.** Descriptive statistics of growth performance, digestibility and feed efficiency, rumen parameter, blood parameter, and faecal parameter of pre-weaning calves supplied with probiotics.

**Additional file 2: Fig. S1.** The flowchart of the search strategy and selection of eligible studies for meta-analysis of the effects of probiotics on pre-weaning dairy calves.

**Additional file 3: Fig. S2.** Risk of bias graph depicting review authors’ judgements about each risk of bias item presented as percentages across all included studies.

**Additional file 4: Fig. S3.** Risk of bias summary depicting authors’ judgements about each risk of bias item for each included study.

## Abbreviations

ADG: Average daily gain
ALB: Albumin
ALP: Alkaline phosphatase
ALT: Alanine aminotransferase
AST: Aspartate aminotransferase
BHBA: Beta-hydroxybutyric acid
BUN: Blood urea nitrogen
BW: Body weight
CI: Confidence interval
FCR: Feed conversion rate
GSH-Px: Glutathione peroxidase
IFNγ: Interferon-γ
IgA: Immunoglobulin A
IGF1: Insulin-like growth factor 1
IgG: Immunoglobulin G
IgM: Immunoglobulin M
LDH: Lactate dehydrogenase
MDA: Malondialdehyde
RMD: Raw mean difference
SDMI: Starter dry matter intake
SMD: Standardized mean difference
SOD: Superoxide dismutase
T-AOC: Total antioxidant capacity
TDMI: Total dry matter intake
VFA: Volatile fatty acids
