# Supplementary material for: A meta-analysis on the effects of probiotics on the performance of pre-weaning dairy calves
Source: J Anim Sci Biotechnol. 2023 Jan 4;14:3. doi: 10.1186/s40104-022-00806-z (PMC9811714; doi:10.1186/s40104-022-00806-z)
Supplement: Supplementary file 1 — Additional file 1: Table S1. Characteristics of the included studies in the meta-analysis. Table S2. Descriptive statistics of growth performance, digestibility and feed efficiency, rumen parameter, blood parameter, and faecal parameter of pre-weaning calves supplied with probiotics. [file 40104_2022_806_MOESM1_ESM.doc]

**Table S1** Characteristics of the included studies in the meta-analysis

| **Study** | **Country** | **Breed** | **Age, d** | ***n*** | **Probiotic** | **Dosage** | **Duration, d** | **Feed** |
| --- | --- | --- | --- | --- | --- | --- | --- | --- |
| Abe et al. (1995) [1] | Japan | Holstein | 1 | 19 | *L. acidophilus, B.thermophilum, E.faecium* | 2.1 × 10^10^ CFU/calf/d | 56 | Milk replacer |
| Abu-Tarboush et al. (1996) [2] | Saudi Arabia | Holstein | 3.5 | 16 | *Lactobacillus acidophilus, Lactobacillus plantarum* | (1) 1.25 g culture/100 kg of milk  (2) 1.85×10^7^/ CFU/L | 84 | Whole milk, milk replacer, starter |
| Agazzi et al. (2014) [3] | Italy | Holstein | 2 | 22 | *L. animalis, L. paracasei, B. coagulans* | 1.8 × 10^10^ CFU/calf/d | 26 | Milk replacer, concentrate |
| Al-Saiady (2010) [4] | Arabia | Holstein | 3 | 16 | *L. acidophilus, L. plantarum* | 1.25 g culture/100 kg of milk |  | Whole milk, milk replacer, starter |
| B. Wang et al. (2018) [5] | China | Holstein | 28 | 10 | *Candida tropicalis* | 5.0 × 10^9^ CFU/calf/d | 28 | Milk replacer, starter |
| Bayatkouhsar et al. (2013) [6] | Iran | Holstein | 4 | 16 | *L. acidophilus, L. rhamnosus, L. casei, L. delbrueckii* | 4.0 × 10^8^ CFU/calf/d | 86 | Whole milk, starter |
| Cruywagenet al. (1996) [7] | South Africa | Holstein | 2 | 40 | *Lactobacillus acidophilus* | 1.0 × 10^8^ CFU/ calf/d | 42 | Milk replacer, starter |
| El-Diastyet al. (2021) [8] | Egypt | Holstein |  | 20 | *Pediococcus* | 2.3×10^7^ CFU/calf/d | 42 | Milk, starter |
| Foditsch et al. (2015) [9] | USA | Holstein | 5 | 287 | *Faecalibacterium prausnitzii* | 1.07 × 10^8^ CFU/calf/d | 14 | Acidified milk, starter |
| Fomenky et al. (2017) [10] | Canada | Holstein | 4.5 | 24 | *Lactobacillus acidophilus, Saccharomyces cerevisiae boulardii* | (1) 7.5 × 10^8^ CFU/L of Milk replacer  (2) 2.5 × 10^8^ CFU/L of Milk replacer | 89 | Milk replacer, starter, hay |
| Frizzo et al. (2008) [11] | Argentina | Holstein | 10 | 24 | *L. casei, L. salivarius, P. acidilactici* | 1 × 10^9^ CFU/kg live weight/d | 35 | Milk replacer, concentrate |
| Frizzo et al. (2010) [12] | Argentina | Holstein | 10 | 16 | *L. casei, L. salivarius, P. acidilactici* | 1 × 10^9^ CFU/kg live weight/d | 35 | Milk replacer, concentrate |
| Gorgulu et al. (2003) [13] | Turkey | Holstein | 3 | 22 | *Lactobacillus plantarum, L. bulgaricus, L. acidophilus, L. mamnsus, Bifidobacterium bifidum, Streptococcus thermophilus, Enterococcus faecium, Aspergillus oryza, and Candida pintolopessi* | 6.16 × 10^8^ CFU/calf/d | 60 | Whole milk, starter, alfalfa |
| Gorka et al. (2021) [14] | Poland | Holstein | 10 | 32 | *Bacillus licheniformis, Bacillus subtilis* | 6.5 × 10^5^ CFU/g of Milk replacer | 50 | Milk replacer, starter |
| Guo et al. (2021) [15] | China | Holstein | 2 | 20 | *Bifidobacterium animalis, Lactobacillus casei, Streptococcus faecalis, Bacillus cerevisiae* | 2.2 × 10^7^ CFU/g  (1) Test1 at 2 g/calf/d  (2) Test2 at 4 g/calf/d  (3) Test3 at 6 g/calf/d | 30 | Milk replacer, starter |
| H. Wang et al. (2021) [16] | China | Holstein | 1 | 32 | *Lactobacillus plantarum, Pediococcus acidilactici, Pediococcus pentosaceus, Bacillus subtilis* | (1) 3.72 × 10^7^ CFU/calf/d  (2) 3.72 × 10^8^ CFU/calf/d | 90 | Milk replacer, starter |
| He et al. (2017) [17] | Canada | Holstein | 1 | 42 | *Saccharomyces cerevisiae var boulardii* | 1.0 × 10^9^ CFU/calf/d | 56 | Milk replacer, starter |
| Higginbotham et al. (1993) [18] | USA | Holstein | 9 | 50 | *L. acidophilus, Streptococcus faecium* | 1.0 × 10^9^ CFU/calf/d | 36 | Milk replacer, starter |
| Hill et al. (2009) [19] | USA | Holstein | 2 | 76 | *Saccharomyces cerevisiae* | 4 g/d of yeast product | 63 | Whole milk, starter |
| Jatkauskas et al. (2010) [20] | Lithuania | Holstein | 6 | 20 | *Enterococcus faecium* | 1.2 × 10^10^ CFU/calf/d | 56 | Fresh milk, skimmed milk, meal feed, hay, grass silage |
| Jennyet al. (1991) [21] | USA | Holstein | 2 | 56 | *Bacillus subtilis, L. acidophilus, L. lactis, B. subtilis* | 1.57 × 10^10^ CFU/calf/d | 42 | Milk replacer, starter |
| Jiang et al. (2020) [22] | China | Holstein | 3 | 24 | *Lactobacillus plantarum* | 1.0 × 10^10^ CFU/calf/d | 56 | Milk, starter |
| Karamzadeh et al. (2020) [23] | Iran | Holstein | 1 | 60 | *Enterococcus faecium, Pediococcus acidilactici, Streptococcus thermophilus, Lactobacillus bulgaricus, Lactobacillus acidophilus, Lactobacillus rhamnosus, Bifidobacterium bifidum* | 1.0 × 10^8^ CFU/calf/d | 28 | Milk, starter |
| Kawakami et al. (2010) [24] | Japan | Holstein | 6 | 8 | Lactic acid bacteria*, S. cerevisiae* | 3.7 × 10^11^ CFU/calf/d | 28 | Milk replacer |
| Kekana et al. (2020) [25] | South Africa | Holstein | 4 | 16 | *L. bavaricus, L. casei, L. rhamnosus, L. coryniformis, L. curvatus, L. sake, S. species, Leuconostoc* | 5.2 × 10^7^ CFU/calf/d | 42 | Whole milk, starter |
| Khaziakhmetov et al. (2020) [26] | Russia | Holstein | 8.5 | 20 | *E. coli, L. acidophilus, Saccharomyces cerevisiae, Bacillus subtilis, Azotobacter vinelandii, Azotobacter chroococcum.* | (1) 10 mL culture  (2) 15 mL culture | 90 | Whole milk, starter, green fodder |
| Kim et al. (2020) [27] | Korea | Holstein | 7 | 18 | *Saccharomyces cerevisiae* | 0.2% hydrolyzed yeast of starter | 42 | Whole milk, starter, grass hay |
| Kong et al. (2019) [28] | China | Holstein | 28 | 24 | *Candida tropicalis* | 5.0 × 10 ^9^ CFU/calf/d | 53 | Milk replacer, starter |
| Laborde (2008) [29] | America | Holstein | 1 | 24 | *Bacillus lichenformis, Bacillus subtilis* | 6.2 × 10^8^ CFU/calf/d | 56 | Milk replacer, starter |
| Le et al. (2017) [30] | Australia | Holstein-Friesians | 28 | 24 | *Bacillus amyloliquefaciens* | 3.16 × 10^8^ CFU/ kg DM | 56 | Whole milk, starter |
| Morrison et al. (2010) [31] | UK | Holstein–Friesian | 5 | 40 | *Streptococcus faecium* | 5.0 × 10 ^9^ CFU/calf/d | 84 | Milk replacer, starter |
| Pinos et al. (2008) [32] | USA | Holstein | 2 | 16 | *Saccharomyces boulardii* | 2.0 × 10^10^ CFU/calf/d | 56 | Whole milk, starter |
| Roodposhti et al. (2012) [33] | Iran | Holstein | 3 | 32 | Protexin | 2.0 × 10^9^ CFU/calf/d | 56 | Whole milk, starter |
| Rychen et al. (2018) [34] | Europe | Holstein | 18 | 80 | *Bacillus subtilis* | 1.0 × 10^9^ CFU/kg of Milk replacer | 56 | Milk replacer, starter |
| Salazar et al. (2019) [35] | Brazil | Holstein | 6 | 20 | *Enterococcus faecium* | 70 mg/kg of starter | 54 | Whole milk, starter |
| Saremi et al. (2003) [36] | Iran | Holstein | 1 | 12 | *Saccharomyces cerevisiae* | 1% yeast of starter | 90 | Milk, starter, alfalfa |
| Seifzadeh et al. (2017) [37] | Iran | Holstein | 5 | 10 | *L. plantarum, L. bulgaricus, L. acidophilus, L. rhamnosus, B. bifidum, S. thermophilus, E. faecium, A. oryzae, C. pintolopesii* | 4.0 × 10^7^ CFU/calf/d | 60 | Whole milk, alfalfa hay |
| Stefańska et al. (2021) [38] | Poland | Holstein-Friesian | 3 | 22 | multi-strain *Lactobacillus* | 2.5 × 10^10^ CFU/calf/d | 53 | Milk replacer, starter |
| Strzetelski et al. (1998) [39] | Poland | Holstein | 7 | 20 | *L. acidophilus, L. casei, L. plantarium, S. faecium* | 4 g probiotics/calf/d | 74 | Milk replacer, concentrate |
| Sun et al. (2010) [40] | China | Holstein | 7 | 12 | *Bacillus subtilis natto* | 1.0 × 10^10^ CFU/calf/d | Weaned when starter intake reached 2% of their weight. | Fresh milk, starter |
| Takemura et al. (2020) [41] | Japan | Holstein | 20 | 24 | *Saccharomyces cerevisiae* | 2.0 × 10^9^ CFU/calf/d | 50 | Whole milk, starter |
| Terré et al. (2015) [42] | Spain | Holstein | 9 | 120 | *Saccharomyces cerevisiae* | 1.0 × 10^9^ CFU/calf/d | 56 | Milk replacer, concentrate |
| Villot et al., (2019) [43] | Canada | Holstein | 6 | 84 | *Saccharomyces cerevisiae boulardii* | 1.0 × 10^10^ CFU/calf/d | 49 | Milk replacer, starter |
| Wu et al., (2021) [44] | Chinese | Holstein | 3 | 20 | *L. acidophilus, B. subtilis, S. cerevisiae* | 7.0 × 10^9^ CFU/g  (1) Test1 at 0.5 g/calf/d  (2) Test2 at 1 g/calf/d  (3) Test3 at 2 g/calf/d | 56 | Milk, starter concentrates |
| Yao et al. (2020) [45] | China | Holstein | 1 | 20 | *Bacillus megaterium* | 5.0 × 10^9^ CFU/calf/d | 28 | Milk, starter, alfalfa |
| Zábranský et al. (2021) [46] | Czech | Holstein | 1 | 80 | *Lactobacillus sporogenes, Enterococcus faecalis, Bifidobacterium bifidum* | 2.05 × 10^8^ CFU/calf/d | 56 | Milk replacer, starter, alfalfa hay |
| Zhang et al. (2016) [47] | China | Holstein | 7 | 16 | *L. plantarum, B. subtilis* | (1) 1.7 × 10^10^ CFU/calf/d  (2) (1.7 × 10^10^ + 1.7 × 10^8^) CFU/calf/d | 75 | Milk replacer, starter, alfalfa |
| Zhang et al. (2017) [48] | China | Holstein | 8 | 8 | *Lactobacillus plantarum* | (1) 1.7 × 10^10^ CFU/calf/d  (2) (1.7 × 10^10^ + 1.7 × 10^8^) CFU/calf/d | 84 | Milk replacer, starter, alfalfa |
| Zhang et al. (2019) [49] | China | Holstein | 12 | 24 | *Lactobacillus rhamnosus* GG | 1.0 × 10^10^ CFU/calf/d | 42 | Milk replacer, starter, alfalfa hay |

**Articles included in this meta-analysis:**

1. Abe F, Ishibashi N, Shimamura S. Effect of administration of bifidobacteria and lactic acid bacteria to newborn calves and piglets. J Dairy Sci. 1995;78:2838-46.

2. Abu-Tarboush HM, Al-Saiady MY, Keir El-Din AH. Evaluation of diet containing *Lactobacilli* on performance, fecal coliform, and *Lactobacilli* of young dairy calves. Anim Feed Sci Technol. 1996;57:39-49.

3. Agazzi A, Tirloni E, Stella S, Maroccolo S, Ripamonti B, Bersani C, et al. Effects of species-specific probiotic addition to milk replacer on calf health and performance during the first month of life. Ann Anim Sci. 2014;14:101-15.

4. Al-Saiady MY. Effect of probiotic bacteria on immunoglobulin G concentration and other blood components of newborn calves. J Anim Vet Adv. 2010;9:604-9.

5. Wang B, Yang CT, Diao QY, Tu Y. The influence of mulberry leaf flavonoids and *Candida tropicalis* on antioxidant function and gastrointestinal development of preweaning calves challenged with *Escherichia coli* O141:K99. J Dairy Sci. 2018;101:6098-108.

6. Bayatkouhsar J, Tahmasebi AM, Naserian AA, Mokarram RR, Valizadeh R. Effects of supplementation of lactic acid bacteria on growth performance, blood metabolites and fecal coliform and *Lactobacilli* of young dairy calves. Anim Feed Sci Technol. 2013;186:1-11.

7. Cruywagen CW, Jordaan I, Venter L. Effect of *Lactobacillus acidophilus* supplementation of milk replacer on preweaning performance of calves. J Dairy Sci. 1996;79:483-6.

8. El-Diasty M, Zayed S, Ouda M, Younes A, Gabr E-S. Effectiveness of dietary pediococcus on growth and some vital biochemical parameters in calves. Zagazig Vet J. 2021;49:222-31.

9. Foditsch C, Pereira RV, Ganda EK, Gomez MS, Marques EC, Santin T, et al. Oral administration of *Faecalibacterium prausnitzii* decreased the incidence of severe diarrhea and related mortality rate and increased weight gain in preweaned dairy heifers. PLoS One. 2015;10:e0145485.

10. Fomenky BE, Chiquette J, Bissonnette N, Talbot G, Chouinard PY, Ibeagha-Awemu EM. Impact of *Saccharomyces cerevisiae boulardii* CNCMI-1079 and *Lactobacillus acidophilus* BT1386 on total lactobacilli population in the gastrointestinal tract and colon histomorphology of Holstein dairy calves. Anim Feed Sci Technol. 2017;234:151-61.

11. Frizzo LS, Bertozzi E, Soto LP, Zbrun MV, Sequeira G, Santina RD, et al. The effect of supplementation with three lactic acid bacteria from bovine origin on growth performance and health status of young calves. J Anim Vet Adv. 2008;7:400-8.

12. Frizzo LS, Soto LP, Zbrun MV, Bertozzi E, Sequeira G, Rodriguez Armesto R, et al. Lactic acid bacteria to improve growth performance in young calves fed milk replacer and spray-dried whey powder. Anim Feed Sci Technol. 2010;157:159-67.

13. Gorgulu M, Siuta A, Ongel E, Yurtseven S, Rustu Kutl H. Effect of probiotic on growing performance and health of calves. Pak J Biol Sci. 2003;6:651-4.

14. Gorka P, Budzinska K, Budzinski W, Jankowiak T, Kehoe S, Kanski J. Effect of probiotic and nucleotide supplementation in milk replacer on growth performance and fecal bacteria in calves. Livest Sci. 2021;250:104556.

15. Guo Y, Li Z, Deng M, Li Y, Liu G, Liu D, et al. Effects of a multi-strain probiotic on growth, health and fecal bacterial flora of neonatal dairy calves. Anim Biosci. 2021;35:204.

16. Wang H, Yu Z, Gao Z, Li Q, Qiu X, Wu F, et al. Effects of compound probiotics on growth performance, rumen fermentation, blood parameters, and health status of neonatal Holstein calves. J Dairy Sci. 2021;105:2190-200.

17. He ZX, Ferlisi B, Eckert E, Brown HE, Aguilar A, Steele MA. Supplementing a yeast probiotic to pre-weaning Holstein calves: feed intake, growth and fecal biomarkers of gut health. Anim Feed Sci Technol. 2017;226:81-7.

18. Higginbotham GE, Bath DLJJoDS. Evaluation of *Lactobacillus* fermentation cultures in calf feeding systems. J Dairy Sci. 1993;76:615-20.

19. Hill SR, Hopkins BA, Davidson S, Bolt SM, Diaz DE, Brownie C, et al. The addition of cottonseed hulls to the starter and supplementation of live yeast or mannanoligosaccharide in the milk for young calves. J Dairy Sci. 2009;92:790-8.

20. Jatkauskas J, Vrotniakiene V. Effects of probiotic dietary supplementation on diarrhoea patterns, faecal microbiota and performance of early weaned calves. Vet Med. 2010;55:494-503.

21. Jenny BF, Vandijk HJ, Collins JA. Performance and fecal flora of calves fed a *Bacillus subtilis* concentrate. J Dairy Sci. 1991;74:1968-73.

22. Jiang X, Xu HJ, Cui ZQ, Zhang YG. Effects of supplementation with *Lactobacillus plantarum* 299v on the performance, blood metabolites, rumen fermentation and bacterial communities of preweaning calves. Livest Sci. 2020;239:104120.

23. Karamzadeh-Dehaghani A, Towhidi A, Zhandi M, Karamzadeh-Dehaghani A, Towhidi A, Zhandi M, et al. Combined effect of probiotics and specific immunoglobulin Y directed against *Escherichia coli* on growth performance, diarrhea incidence, and immune system in calves. Animal. 2020;15:100124.

24. Kawakami S-I, Yamada T, Nakanishi N, Cai Y. Feeding of lactic acid bacteria and yeast on growth and diarrhea of Holstein calves. J Anim Vet Adv. 2010;9:1112-4.

25. Kekana TW, Nherera-Chokuda VF, Baloyi JJ, Muya CM. Immunoglobulin G response and performance in Holstein calves supplemented with garlic powder and probiotics. S Afr J Anim Sci. 2020;50:264-71.

26. Khaziakhmetov F, Khabirov A, Tagirov K, Avzalov R, Tsapalova G, Basharov A. The influence of "Stimix Zoostim" and "Normosil" probiotics on fecal microflora, hematologic indicators, nutrient digestibility, and growth of mother-bonded calves. Vet World. 2020;13:1091-7.

27. Kim ET, Lee HG, Kim DH, Son JK, Kim B-W, Joo SS, et al. Hydrolyzed yeast supplementation in calf starter promotes innate immune responses in Holstein calves under weaning stress condition. Animals. 2020;10:1468.

28. Kong L, Yang C, Dong L, Diao Q, Si B, Ma J, et al. Rumen fermentation characteristics in pre-and post-weaning calves upon feeding with mulberry leaf flavonoids and *Candida tropicalis* individually or in combination as a supplement. Animals. 2019;9:990.

29. Laborde J. Effects of probiotics and yeast culture on rumen development and growth of dairy calves. 2008.

30. Le OT, Dart PJ, Harper K, Zhang D, Schofield B, Callaghan MJ, et al. Effect of probiotic *Bacillus amyloliquefaciens* strain H57 on productivity and the incidence of diarrhoea in dairy calves. Anim Prod Sci. 2017;57:912-9.

31. Morrison SJ, Dawson S, Carson AF. The effects of mannan oligosaccharide and *Streptococcus faecium* addition to milk replacer on calf health and performance. Livest Sci. 2010;131:292-6.

32. Pinos-Rodríguez JM, Robinson PH, Ortega ME, Berry SL, Mendoza G, Bárcena R. Performance and rumen fermentation of dairy calves supplemented with *Saccharomyces cerevisiae*1077 or *Saccharomyces boulardii* 1079. Anim Feed Sci Technol. 2008;140:223-32.

33. Roodposhti PM, Dabiri N. Effects of probiotic and prebiotic on average daily gain, fecal shedding of *Escherichia coli*, and immune system status in newborn female calves. Asian-Australas J Anim Sci. 2012;25:1255-61.

34. Rychen G, Aquilina G, Azimonti G, Bampidis V, Bastos MdL, Bories G, et al. Safety and efficacy of *Bacillus subtilis* DSM 28343 as a feed additive for calves for rearing EFSA J. 2018;16:e05220.

35. Salazar LFL, Nero LA, Campos-Galvao MEM, Cortinhas CS, Acedo TS, Tamassia LFM, et al. Effect of selected feed additives to improve growth and health of dairy calves. PLoS One. 2019;14:e0216066.

36. Saremi B, Naserian A. Improvement of the growth and performance of Holstein neonatal calves receiving the microbial additive *Saccharomyces cerevisiae*. J Dairy Sci. 2003;86:3809.

37. Seifzadeh S, Aghjehgheshlagh FM, Abdibenemar H, Seifdavati J, Navidshad B. The effects of a medical plant mix and probiotic on performance and health status of suckling Holstein calves. Ital J Anim Sci. 2017;16:44-51.

38. Stefańska B, Sroka J, Katzer F, Goliński P, Nowak W. The effect of probiotics, phytobiotics and their combination as feed additives in the diet of dairy calves on performance, rumen fermentation and blood metabolites during the preweaning period. Anim Feed Sci Technol. 2021;272.

39. Strzetelski JA, Kowalczyk J, Krawczyk K. Effect of various probiotics on calf performance. J Anim Feed Sci. 1998;7:241-4.

40. Sun P, Wang JQ, Zhang HT. Effects of *Bacillus subtilis natto* on performance and immune function of preweaning calves. J Dairy Sci. 2010;93:5851-5.

41. Takemura K, Shingu H, Ikuta K, Sato S, Kushibiki S. Effects of *Saccharomyces cerevisiae* supplementation on growth performance, plasma metabolites and hormones, and rumen fermentation in Holstein calves during pre- and post-weaning periods. Anim Sci J. 2020;91:e13402.

42. Terré M, Maynou G, Bach A, Gauthier M. Effect of *Saccharomyces cerevisiae* CNCM I-1077 supplementation on performance and rumen microbiota of dairy calves. Prof Anim Sci. 2015;31:153-8.

43. Villot C, Ma T, Renaud DL, Ghaffari MH, Gibson DJ, Skidmore A, et al. *Saccharomyces cerevisiae boulardii* CNCM I-1079 affects health, growth, and fecal microbiota in milk-fed veal calves. J Dairy Sci. 2019;102:7011-25.

44. Wu Y, Wang L, Luo R, Chen H, Nie C, Niu J, et al. Effect of a multispecies probiotic mixture on the growth and incidence of diarrhea, immune function, and fecal microbiota of pre-weaning dairy calves. Front Microbiol. 2021;12:681014.

45. Yao J, Wang LL, Zhang WJ, Liu MJ, Niu JL. Effects of *Bacillus megaterium* on growth performance, serum biochemical parameters, antioxidant capacity, and immune function in suckling calves. Open Life Sci. 2020;15:1033-41.

46. Zábranský L, Poborská A, Malá G, Gálik B, PETRÁŠKOVÁ E, KERNEROVÁ N, et al. Probiotic and prebiotic feed additives in calf nutrition. J Centr Eur Agric. 2021;22:14-8.

47. Zhang R, Zhou M, Tu Y, Zhang NF, Deng KD, Ma T, et al. Effect of oral administration of probiotics on growth performance, apparent nutrient digestibility and stress-related indicators in Holstein calves. J Anim Physiol Anim Nutr. 2016;100:33-8.

48. Zhang R, Dong X, Zhou M, Tu Y, Zhang N, Deng K, et al. Oral administration of *Lactobacillus plantarum* and *Bacillus subtilis* on rumen fermentation and the bacterial community in calves. Anim Sci J. 2017;88:755-62.

49. Zhang L, Jiang X, Liu X, Zhao X, Liu S, Li Y, et al. Growth, health, rumen fermentation, and bacterial community of Holstein calves fed *Lactobacillus rhamnosus* GG during the preweaning stage. J Anim Sci. 2019;97:2598-608.

**Table S2** Descriptive statistics of growth performance, digestibility and feed efficiency, rumen parameter, blood parameter, and faecal parameter of pre-weaning calves supplied with probiotics

| **Items** | | ***n*** | **Mean** | **SD** | **Minimum** | **Maximum** | **Median** |
| --- | --- | --- | --- | --- | --- | --- | --- |
| **Growth performance** | |  |  |  |  |  |  |
|  | Wither height, cm | 26 | 82.98 | 4.49 | 70 | 91.6 | 82.49 |
|  | Heart girth, cm | 25 | 92.73 | 8.77 | 83.9 | 115.4 | 91.71 |
|  | Hip width, cm | 7 | 21.51 | 2.06 | 19.4 | 24.5 | 21.5 |
|  | Hip height, cm | 14 | 88.25 | 4.54 | 80.8 | 96.6 | 86.35 |
|  | Body length, cm | 11 | 86.24 | 11.28 | 65.27 | 102.7 | 85.6 |
|  | Body weight, kg | 62 | 76.80 | 25.76 | 38.15 | 136.65 | 74.65 |
|  | ADG, g/d (Average daily gain) | 79 | 553.60 | 230.70 | 22.6 | 1149 | 570 |
| **Digestibility and Feed efficiency** | |  |  |  |  |  |  |
|  | OM Digestibility, % (Organic matter) | 8 | 87.68 | 3.94 | 82.4 | 94 | 87.5 |
|  | DM Digestibility, % (Dry matter) | 8 | 85.94 | 4.63 | 79.1 | 93 | 85.77 |
|  | EE Digestibility, % (Ether extract) | 5 | 86.48 | 10.12 | 73.3 | 97 | 93.5 |
|  | CP Digestibility, % (Crude protein) | 7 | 84.62 | 3.71 | 79.7 | 92 | 84.51 |
|  | NDF Digestibility, % (Neutral detergent fiber) | 2 | 63.75 | 7.75 | 56 | 71.5 | 63.75 |
|  | ADF Digestibility, % (Acid detergent fiber) | 3 | 58.58 | 2.89 | 55.2 | 62.27 | 58.26 |
|  | Starter dry matter intake, kg/d (SDMI) | 42 | 3.10 | 7.88 | 0.1 | 30.82 | 0.57 |
|  | Total dry matter intake, kg/d (TDMI) | 47 | 1.03 | 0.36 | 0.07 | 1.67 | 1 |
|  | FCR, kg/kg (TDMI: ADG) | 35 | 2.25 | 0.74 | 0.60 | 3.51 | 2.43 |
| **Rumen parameter** | |  |  |  |  |  |  |
|  | pH (Rumen pH) | 20 | 6.03 | 0.45 | 5.4 | 7 | 5.90 |
|  | MCP, mg/dL (Microbial protein) | 2 | 183.39 | 14.92 | 168.47 | 198.3 | 183.39 |
|  | NH_3_, mmol/L | 17 | 7.43 | 4.31 | 3.33 | 16.4 | 5.42 |
|  | Total VFA, mmol/L | 13 | 73.10 | 18.79 | 49.99 | 119 | 64.9 |
|  | Acetate, mmol/L | 10 | 38.57 | 9.47 | 22.4 | 60.12 | 36.95 |
|  | Propionate, mmol/L | 12 | 25.38 | 9.35 | 9.7 | 38.6 | 24.67 |
|  | Butyrate, mmol/L | 15 | 6.50 | 4.72 | 0.4 | 14.57 | 5.11 |
|  | Valerate, mmol/L | 15 | 2.37 | 2.54 | 0.43 | 8.31 | 1.17 |
| **Blood parameter** | |  |  |  |  |  |  |
|  | **Biochemical indexes** |  |  |  |  |  |  |
|  | ALP, U/L (Alkaline phosphatase) | 8 | 145.37 | 86.63 | 30.3 | 267.75 | 166.43 |
|  | ALB, g/L (Albumin) | 9 | 30.20 | 3.32 | 25.85 | 35.8 | 29.4 |
|  | ALT, U/L (Alanine aminotransferase) | 7 | 13.75 | 6.87 | 6.34 | 26.4 | 14.22 |
|  | AST, U/L (Aspartate aminotransferase) | 8 | 46.73 | 15.88 | 31.6 | 86 | 43.73 |
|  | BHBA, mmol/L (β-hydroxybutyric acid) | 7 | 0.30 | 0.09 | 0.14 | 0.42 | 0.29 |
|  | BUN, mmol/L (Blood urea nitrogen) | 14 | 3.56 | 1.22 | 2.11 | 6.32 | 3.43 |
|  | Glu, mmol/L (Glucose) | 20 | 4.15 | 1.39 | 0.58 | 5.75 | 4.40 |
|  | TP, g/L (Total protein) | 20 | 57.98 | 8.67 | 44 | 86.2 | 56.92 |
|  | TC, mmol/L (Total cholesterol) | 12 | 2.17 | 0.56 | 1.32 | 3.03 | 2.16 |
|  | TG, mmol/L (Triglyceride) | 5 | 0.27 | 0.08 | 0.15 | 0.37 | 0.3 |
|  | LDH, U/L (Lactate dehydrogenase) | 6 | 620.13 | 197.21 | 283 | 819.09 | 659.01 |
|  | **Immune indices** |  |  |  |  |  |  |
|  | IgA, g/L (Immunoglobulin A) | 24 | 3.30 | 1.05 | 0.73 | 4.92 | 3.42 |
|  | IgG, g/L (Immunoglobulin G) | 26 | 18.14 | 13.12 | 4.24 | 73.35 | 14.49 |
|  | IgM, g/L (Immunoglobulin M) | 25 | 2.29 | 0.84 | 0.83 | 3.7 | 2.13 |
|  | IGF-1, ng/mL (Insulin-like growth factor 1) | 7 | 63.70 | 44.10 | 32.2 | 167.7 | 46.8 |
|  | IFN-γ, pg/ml (Interferon-γ) | 4 | 50.34 | 14.96 | 34.39 | 67.65 | 49.65 |
|  | **Antioxidant indices** |  |  |  |  |  |  |
|  | MDA, nmol/mL (Malondialdehyde) | 5 | 4.39 | 0.75 | 2.99 | 5.06 | 4.49 |
|  | GSH-P*x*, U/mL (Glutathione peroxidase) | 5 | 141.85 | 27.97 | 88.1 | 167.66 | 148.87 |
|  | SOD, U/mL (Superoxide dismutase) | 9 | 125.73 | 59.57 | 53.51 | 212.4 | 161.2 |
|  | T-AOC, U/mL (Total antioxidant capacity) | 8 | 8.60 | 4.66 | 3.85 | 16.2 | 5.75 |
| **Faecal parameter** | |  |  |  |  |  |  |
|  | Fecal score | 25 | 1.42 | 0.39 | 0.62 | 2.39 | 1.5 |
|  | Faecal bacteria counts, CFU/g | 12 | 12.69 | 9.39 | 7.04 | 39.3 | 8.56 |
|  | coliform, CFU/g | 16 | 7.10 | 3.09 | 2.59 | 16.2 | 7.73 |
|  | *Lactobacilli*, log_10_CFU/g | 23 | 6.48 | 3.46 | 1.50 | 16.2 | 7.5 |
|  | *Streptococcus*, CFU/g | 6 | 11.65 | 9.77 | 5.75 | 33 | 6.76 |
