# Supplementary material for: A meta-analysis on the effects of probiotics on the performance of pre-weaning dairy calves
Source: J Anim Sci Biotechnol. 2023 Jan 4;14:3. doi: 10.1186/s40104-022-00806-z (PMC9811714; doi:10.1186/s40104-022-00806-z)
Supplement: Supplementary file 2 — Additional file 2: Fig. S1. The flowchart of the search strategy and selection of eligible studies for meta-analysis of the effects of probiotics on pre-weaning dairy calves. [file 40104_2022_806_MOESM2_ESM.doc]

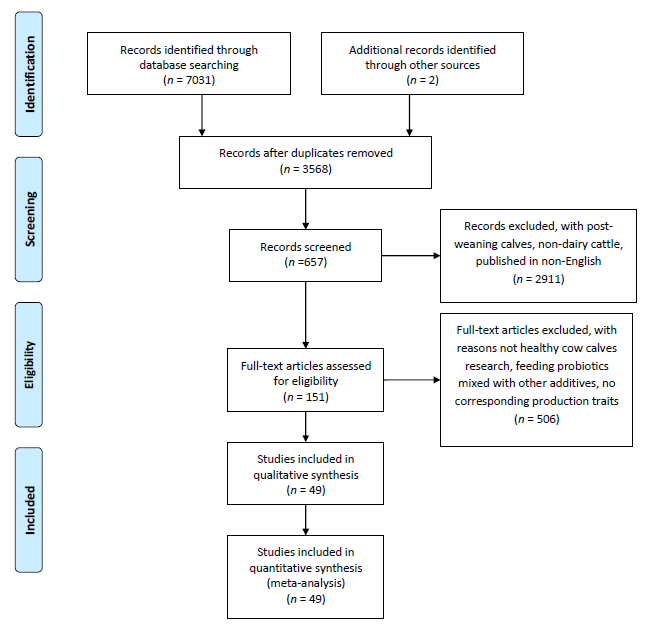


**Fig. S1** The flowchart of the search strategy and selection of eligible studies for meta-analysis of the effects of probiotics on pre-weaning dairy calves
