# Supplementary material for: A meta-analysis on the effects of probiotics on the performance of pre-weaning dairy calves
Source: J Anim Sci Biotechnol. 2023 Jan 4;14:3. doi: 10.1186/s40104-022-00806-z (PMC9811714; doi:10.1186/s40104-022-00806-z)
Supplement: Supplementary file 3 — Additional file 3: Fig. S2. Risk of bias graph depicting review authors’ judgements about each risk of bias item presented as percentages across all included studies. [file 40104_2022_806_MOESM3_ESM.docx]

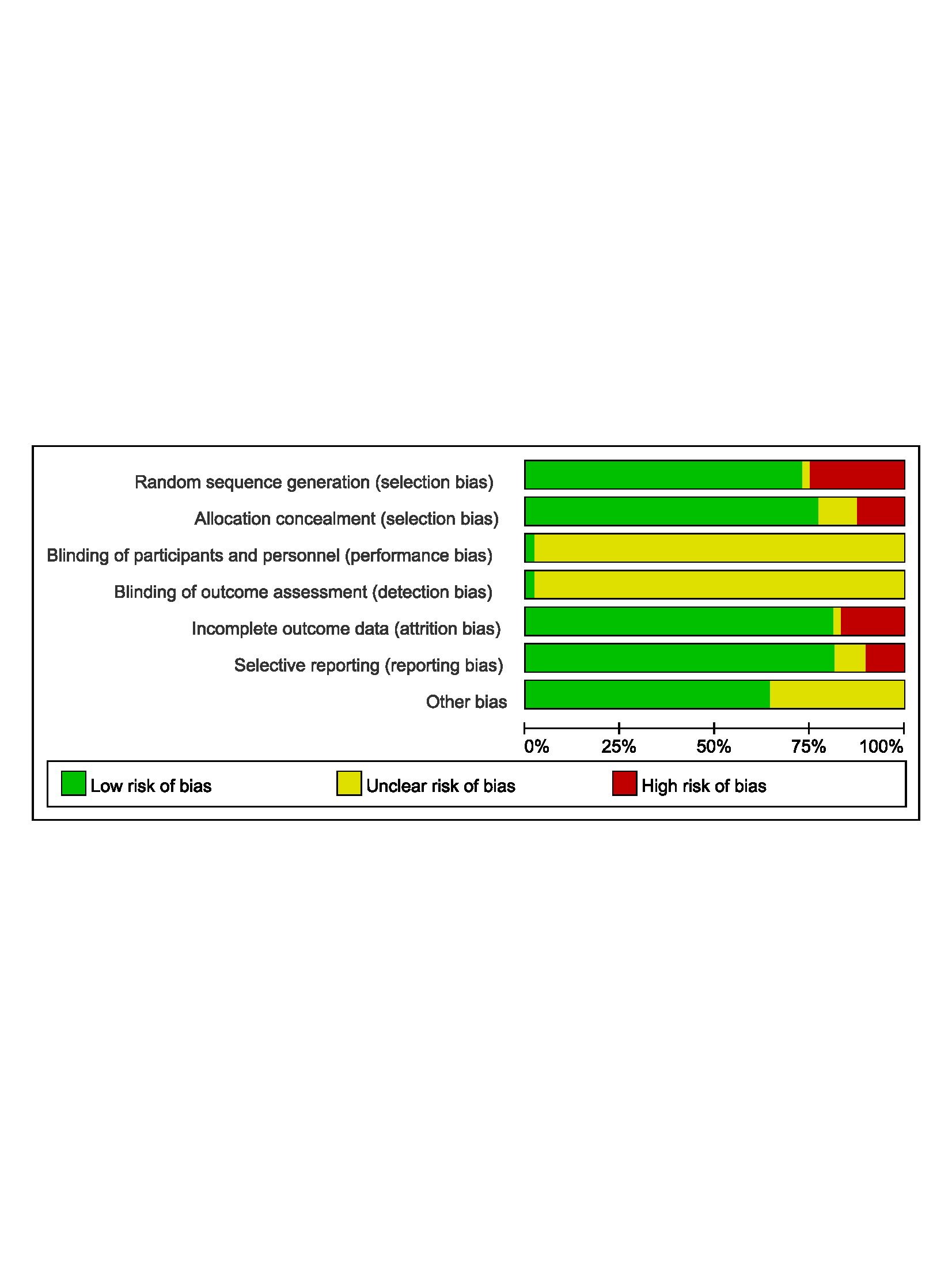


**Fig. S2** Risk of bias graph depicting review authors’ judgements about each risk of bias item presented as percentages across all included studies
