# Supplementary material for: A meta-analysis on the effects of probiotics on the performance of pre-weaning dairy calves
Source: J Anim Sci Biotechnol. 2023 Jan 4;14:3. doi: 10.1186/s40104-022-00806-z (PMC9811714; doi:10.1186/s40104-022-00806-z)
Supplement: Supplementary file 4 — Additional file 4: Fig. S3. Risk of bias summary depicting authors’ judgements about each risk of bias item for each included study. [file 40104_2022_806_MOESM4_ESM.doc]

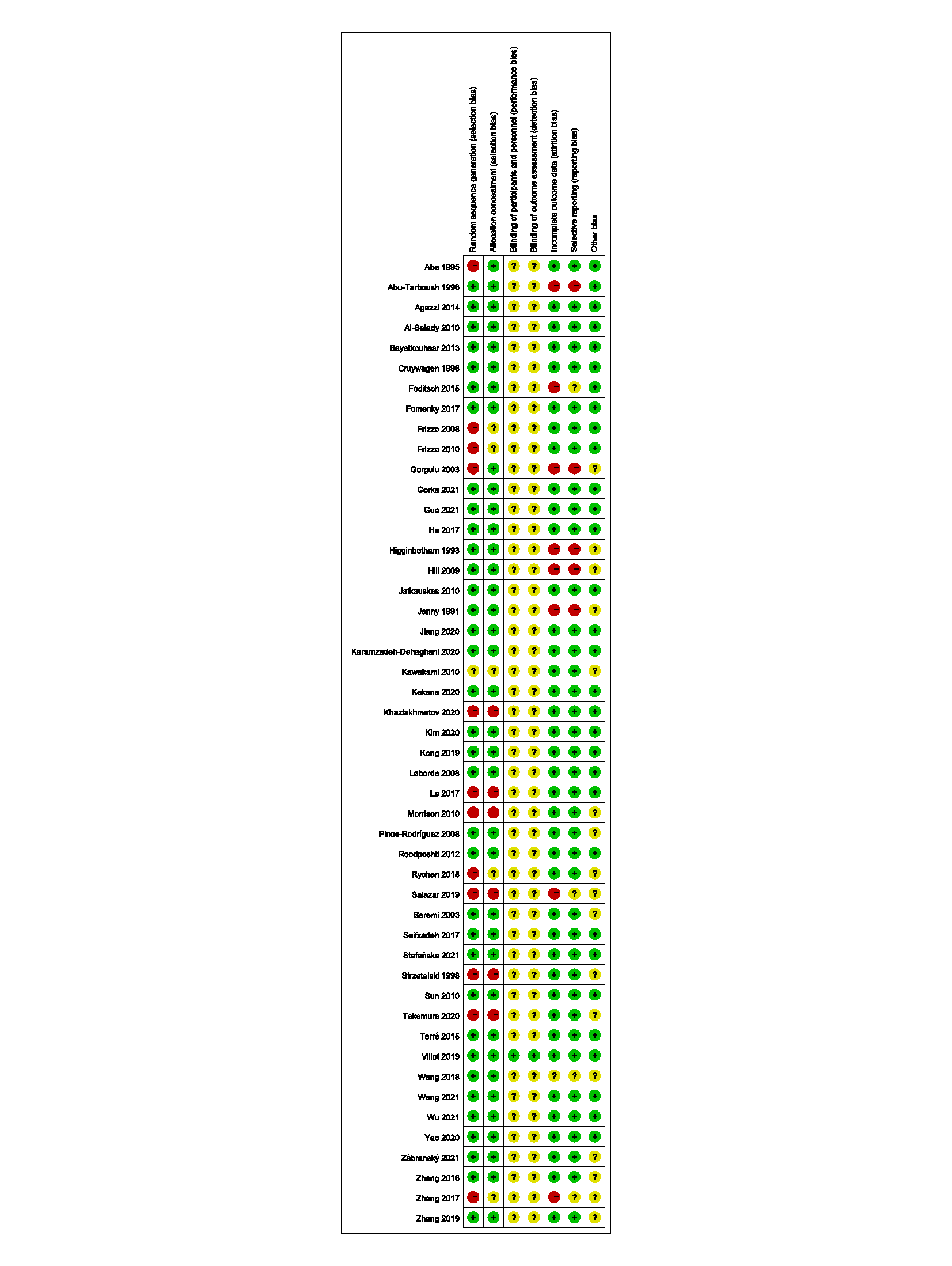


**Fig. S3** Risk of bias summary depicting authors’ judgements about each risk of bias item for each included study
